# The introduction of a value‐based reimbursement programme—Alignment and resistance among healthcare providers

**DOI:** 10.1002/hpm.3574

**Published:** 2022-09-15

**Authors:** Thérèse Eriksson, Lars‐Åke Levin, Ann‐Charlotte Nedlund

**Affiliations:** ^1^ Division of Society and Health (SH) Department of Health, Medicine and Caring Sciences (HMV) Linköping University Linkoping Sweden

**Keywords:** bundled payment, institutional change, neo‐institutional theory, pay‐for‐performance, reimbursement, value‐based reimbursement

## Abstract

Reimbursement programmes are used to manage care through financial incentives. However, their effects are mixed and the programmes can motivate behaviour that goes against professional values. Value‐based reimbursement programmes may better align professional values with financial incentives. The aim of this study is to analyse if and how healthcare providers adapt their practices to a value‐based reimbursement programme that combines bundled payment with performance‐based payment. Forty‐one semi‐structured interviews were conducted with representatives from healthcare providers within spine surgery in Sweden. Data were analysed using thematic analysis with an abductive approach and a conceptual framework based on neo‐institutional theory. Healthcare providers were positive to the idea of a value‐based reimbursement programme. However, during its introduction it became evident that some aspects were easier to adapt to than others. The bundled payment provided a more comprehensive picture of the patients' needs but to an increased administrative burden. Due to the financial impact of the bundled payment, healthcare providers tried to decrease the amount of post‐discharge care. The performance‐based payment was appreciated. However, the lack of financial impact and transparency in how the payment was calculated caused providers to neglect it. Healthcare providers adapted their practices to, but also resisted aspects of the value‐based reimbursement programme. Resistance was mainly caused by lack of understanding of how to interpret and act on new information. Providers had to face unfamiliar situations, which they did not know how to handle. Better IT‐facilitation and clearer definition of related care is needed to strengthen the value‐based reimbursement programme among healthcare providers. A value‐based reimbursement programme seems to better align professional values with financial incentives.

## Highlights

- The idea of value‐based reimbursement was in line with professional values.
- The bundled payment increased the willingness to collaborate.
- The performance‐based payment based on Patient reported outcome measures was too complex to understand.
- Lack of support from purchaser and insufficient IT‐systems caused resistance.

## 1 INTRODUCTION

According to economic theory, financial incentives are powerful means to shape the behaviour of actors in any market, including the health care market. The reach and limits of financial incentives in health care has been widely debated for decades. Some argue that it increases efficient use of scarce resources,^1^ while others voice that it leads to unintended and unethical outcomes.^2^ Studies have shown that financial incentives can be an effective way to affect physician behaviour.^3^, ^4^, ^5^ However, the incentives do not always have the intended effects.^6^, ^7^, ^8^, ^9^ The use of financial incentives has for example, been criticised for increasing production disregarding the quality of care^10^ and may dampen the autonomy among physicians^11^, ^12^ who feel obliged to perform certain activities in order to get reimbursed. A study investigating physicians' perceptions of financial aspects shows that physicians commonly view financial accountability as something that comes at the expense of autonomy, and that high levels of autonomy among physicians is a pre‐requisite for achieving high quality of care.^11^ On the other hand, decreased professional autonomy may increase the control of the purchaser and therefore something positive for them.

Reimbursement programmes are commonly used to generate financial incentives with the ambition of aligning the objectives of healthcare providers and the purchaser.^13^, ^14^ Thus, the incentive design in the programme must take into account the multiple agency connections (e.g., the provider as agent to both patient and purchaser) inherent in the reimbursement for health services.^15^ Poorly designed reimbursement programmes may lead to undesired behaviour and inefficient activities that decrease the legitimacy of financial incentives as an instrument to manage care. Therefore, it is also important to take the context into account, in which the reimbursement programme is introduced since the reimbursement programme alone may not be enough to affect daily operations.^16^

Value‐based reimbursement programmes (VBRP) focus on activities that generate value through quality enhancing, but also cost constraining, incentives.^17^ Surgical procedures have been considered suitable for value‐based reimbursement because of the discrete beginning and end of a care episode. Further, the variation in recommendations in clinical guidelines^18^ of spine surgery makes it suitable for a value‐based reimbursement since the reimbursement level is conditioned on the outcome of the surgery. Hence, the provider must assess whether the patient will improve enough to outweigh the cost of performing the surgery. The purchaser put the financial responsibility on the provider instead of paying for the service no matter the quality.

The regional public health authority, Region Stockholm, is responsible of providing healthcare to 2.4 million people.^19^ Region Stockholm introduced a value‐based reimbursement programme (STHLM‐VBRP) within elective spine surgery in 2013. The design of the programme is based upon the thoughts of value‐based healthcare (VBHC), first outlined by Porter and Teisberg in 2006,^20^ and combines bundled payment with performance‐based payment (also known as pay‐for‐performance, P4P) in a unique design. The bundled payment extends the clinical episode to 1 year after surgery, a longer period compared to other programmes^21^, ^22^; and the measure used for the performance‐based payment is how much pain the patient experiences 1 year after surgery. The bundled payment extends providers' financial responsibility to incentivise coordination of care and to avoid overuse. The performance‐based payment conditions the reimbursement to the outcome of the surgery and aims to enhance and sustain quality by rewarding high‐performing providers. In this paper, we sought to contribute to the empirical value of neo‐institutional theory, by interviewing representatives from healthcare providers on how regulative changes affect daily operations within elective spine surgery in Stockholm, Sweden.

The aim of this study is to analyse if and how healthcare providers adapt their practices to a value‐based reimbursement programme that combines bundled payment with performance‐based payment. In particular, we investigate the following research questions: How do healthcare staff experience and respond to the financial incentives the STHLM‐VBRP entail? How can these experiences be understood from the perspective of neo‐institutional theory in terms of alignment and resistance to the contractual changes the STHLM‐VBRP imposed?

## 2 UNDERSTANDING REGULATIVE CHANGES FROM THE PERSPECTIVE OF INSTITUTIONAL THEORY

The implementation of VBRP varies between, but also within, different healthcare systems.^23^ In the US, focus has been on moving away from fee‐for‐service,^24^ whereas publicly financed healthcare systems in Europe mostly have focused on coordinating care among providers.^25^ Thus, the introduction of the value‐based reimbursement programme (VBRP) does not happen in a vacuum. This may seem obvious, but many evaluations overlook contextual factors when assessing an intervention.^5^, ^26^, ^27^ Organisations cannot be fully understood in isolation from the external influences that arise from a wider contextual perspective.^28^ Consequently, institutional theory provides suitable frameworks for examining the nature of external demands and the behaviour of organisations. We use an approach to new‐institutional theory based on Scott's conceptual framework.^28^ According to Scott, institutions consist of regulative, normative and cultural‐cognitive pillars that in relation to each other has stabilising and meaning‐making properties.

The regulative pillar refers to the practice of rule‐setting, monitoring, sanctioning and incentivising. It comprises formal legislation but also less formal rule making. Instrumentalism is very central within the regulative pillar, that is, individuals conform to laws and rules because they seek rewards or wish to avoid sanctions. Hence, focus on the regulative pillar sheds light on the more formalised control systems. The normative pillar encompasses values and norms. Values refer to conceptions of the preferred or the desirable, whereas norms refer to the scripts for how to reach the desirable goals and what means are legitimate in attaining them.^28^ A focus on the normative pillar emphasises the stabilising influence of social beliefs and norms, both internalised and imposed by others and highlights the ‘moral roots’ of behaviour and institutions. The cultural‐cognitive pillar refers to the processes and frameworks of the shared perception, which enable sense making when meeting the ‘external world of stimuli’.^28^ The cultural‐cognitive pillar emphasises features of shared understanding, professional ideologies, cognitive frames or sets of collective meanings. These aspects condition how actors interpret and respond to the world around them. A focus on the cultural‐cognitive pillar sheds light on how knowledge is constructed and codified in models, assumptions and schemas, to what extent it informs and constrain behaviour. Confusion among actors usually indicates lack of support from the cultural‐cognitive pillar because they do not know how to process or interpret information.

Institutions are more robust when the regulative, normative and cultural‐cognitive pillars are aligned and reinforce each other. Changes in one of the pillars may cause misalignment and resistance, thus weakening the institution if the pillars motivate different behaviours. However, institutions tend to converge over time and institutional theory considers misalignment of pillars as a catalyst for change.^28^

The framework by Scott provides a structure to our analysis by focussing on the respective pillar, but also their interrelationship. The introduction of the STHLM‐VBRP imposed contractual changes regarding the provision of elective spine surgery in Region Stockholm and can thus be regarded as regulative. This change may work as a catalyst for institutional change if the introduction causes a misalignment between the pillars. If healthcare providers resist to institutional elements imposed by the VBRP, these elements will not be institutionalised. If healthcare providers align to the institutional elements imposed by the VBRP, the elements will be institutionalised. By analysing all three pillars, we intend to provide a deeper understanding on how and why certain aspects of the new reimbursement programme are institutionalised or not.

## 3 THE CASE OF THE VALUE‐BASED REIMBURSEMENT PROGRAMME

The Swedish healthcare system is publicly financed with universal coverage. In Sweden there are 21 regions that are responsible for the provision and financing of healthcare, mainly through tax revenues. Since the regions are responsible for both provision and financing, they can be considered as both commissioning and purchasing organisations. As a commissioner, the region decides under what conditions healthcare organisations may provide care in the region. As a purchaser, the region pays for the healthcare consumed by the inhabitants within the region. In our article, Region Stockholm will synonymously be referred to as the purchaser.

To receive public funding, private healthcare providers need accreditation by establishing a commissioning contract with the region in which they wish to deliver care. This is done either through the Public Procurement Act^29^ or through the Freedom of Choice Act^30^ (known as Patient Choice within healthcare), two different market‐oriented solutions. Under the Public Procurement Act, healthcare providers are permitted a certain volume each year to a negotiated price, specific to each healthcare provider. Whereas Patient Choice is a contract that usually have no restriction on volume but with a set price, making providers compete based on quality and ultimately the patients' choice, a requirement for VBHC.^17^ Patient Choice entails a continuous commissioning contract between the purchaser and healthcare providers, instead of a recurring procurement process.

In 2013, Region Stockholm transitioned to accredit healthcare providers through Patient Choice instead of the Public Procurement within elective spine surgery. An elective surgery is scheduled in advance and does not involve an emergency. It was also decided that Patient Choice for elective spine surgery should entail a value‐based reimbursement programme. This reimbursement programme will be referred to as the Stockholm value‐based reimbursement programme (STHLM‐VBRP). Private healthcare providers in Region Stockholm performed most of the elective surgeries, both before and after the introduction of Patient Choice with the STHLM‐VBRP.

The STHLM‐VBRP combines bundled payment with performance‐based payment, adjusted for patient characteristics. When the surgical procedure is registered, the healthcare provider receives a **prospective payment,** which includes the bundled payment and the expected performance‐based payment. The prospective payment is adjusted for age, gender, comorbidity level and surgery that covers more than two levels of the spine. The idea is to limit differences in financial risk between patients to promote need‐based healthcare. Failing to adjust for case‐mix when designing a reimbursement programme leads to an increased risk of ‘cherry picking’, that is, providers avoid clinically complicated patients to the benefit of healthier patients. However, patients with a high potential risk of needing intensive care are not covered by the commissioning contract and must be surgically treated at a hospital with access to an intensive care unit.

The **bundled payment** should cover the individual patient's healthcare utilisation related to the spine surgery (e.g., potential complications, reoperation, rehabilitation), for the full care episode of 1 year. That means that the bundled payment includes the patient's rehabilitation, primary care, speciality care and hospital care provided by external healthcare providers (i.e., not the provider that performed the surgery). The provider that performs the surgery receives an invoice from the purchaser if an external healthcare provider treats their patient after the surgery. Hence, the bundled payment is a multi‐organisational bundle of service. The bundled payment should stimulate an effective and integrated care chain by using a fixed payment to the provider for all services provided during the entire care episode.

The **performance‐based payment** is based on the outcome measure Global Assessment (GA). The measure is a retrospective transition question asked 1 year after surgery (‘How is your back/leg pain today compared to before the surgery?’).^31^ The patient can choose between six response options (pain free, much better, somewhat better, unchanged, worse, did not have pain before the surgery). The registration of GA is administered and managed by the national quality registry for spine surgery in Sweden, Swespine.^32^, ^33^ The expected performance‐based payment that is included in the prospective payment is based on historical outcomes of GA, adjusted for patient characteristics. If the level of pain of the patient turns out better than expected 1 year after surgery, the healthcare provider receives an additional payment. If the level of pain turns out worse than expected the healthcare provider has to repay money to Region Stockholm. Hence, the size of the adjustment depends on the discrepancy between the actual and the expected outcome. A provider cannot receive a positive performance adjustment by performing surgery on a patient that is expected to be pain free 1 year after surgery, simply because that patient cannot get any better. On the other hand, a patient that according to historical outcomes is expected to experience a somewhat better pain will generate a positive adjustment if the actual outcome is pain free. The idea is that the performance‐based payment should give financial incentives to investigate further, what can be done to improve the pain 1 year after surgery. Thus, performance‐based payment is a complement to the bundle payment, to avoid healthcare providers stinting on necessary care since it may negatively affect the pain patients experience. However, healthcare providers cannot perceive the full size of the performance‐based payment since it is included in the prospective payment; they only perceive the adjustment if the actual outcome deviates from the expected.

## 4 STUDY DESIGN AND METHODS

We conducted a systematic comparison of case studies, using respondents' own reports since neo‐institutional analysis assumes that institutions are, in effect, manifested through individuals' attitudes, beliefs and motivation. The case studies of interest were healthcare providers accredited within elective spine surgery and reimbursed based on the STHLM‐VBRP.

At the time of the introduction of the STHLM‐VBRP, there were three accredited healthcare providers in Region Stockholm. A fourth provider was accredited in 2017. All of the providers were private and for‐profit, one clinic was a professional partnership whereas the other three clinics were part of a larger healthcare organisation (however, some had a history of being a cooperative/professional partnership). Two providers were located in Stockholm city, one in a Stockholm suburb and the fourth in a neighbouring region (Region Sörmland). Despite being located in different regions, all healthcare providers were providing care under the same contractual conditions after the introduction of the STHLM‐VBRP.

An interview guide was designed based on the structure of the reimbursement programme. The interview guide was designed as an aide‐memoire,^34^ to ensure that all aspects were covered but still allowing for the respondent to talk freely about the topics. To recruit respondents for interviews, we used a purposive sampling approach^35^ in dialogue with the respective managers at the four clinics. We wanted the respondents to reflect the heterogeneity among staff, thus both clinically active and administrative staff were included from different professions, to attain a more comprehensive perspective. By interviewing both staff and clinicians we could reflect the potential different contextual factors and consequences of the reimbursement programme. All staff were employed by the healthcare provider and their salary was not affected by the new reimbursement programme. Before commencing the fieldwork, we obtained ethical approval (2015/94‐31) from the regional board of ethics in Linköping, as well as a signed consent to participate from each respondent. The respondents were also informed that each interview was estimated to last between 30 and 60 min.

We conducted semi‐structured face‐to‐face interviews with representatives from all four accredited healthcare providers at respective spine surgery clinics. The interviews were carried out in two waves, May 2015–May 2016 and June–September 2017. The interviews were carried out in two waves to cover any potential time factor affecting how respondents experienced the reimbursement programme. For the second wave, our first option was to interview the same respondents, but because of misaligned schedules and certain staff turnover this was not always possible. In total, 41 respondents were interviewed, see Table 1. Seven respondents were interviewed in both the first and the second wave, thus 34 unique respondents were interviewed. Three interviews were conducted with two respondents at the same time after a query from the respondents. Two interviews were conducted over the telephone, both in the second wave, with respondents who had already been interviewed face‐to‐face during the first wave. To make the respondent feel comfortable in the situation, each interview started with more general questions about the respondent's profession and responsibilities.^35^ Each interview lasted between 20 and 60 min. There was some variation in length of the interviews because respondents had been involved and affected by the reimbursement programme to a varying degree. However, each interview started by checking available time in order to adjust the disposition to the topic. The interviews were carried out in Swedish. All but one interview were audio recorded and transcribed verbatim in Swedish.

**TABLE 1:** Represented professions and functions among the respondents

	First wave	Second wave	Total
Profession			
Spine surgeon	4	6	10
Registered nurse	7	9	16
Physiotherapist	1	4	5
Accountant/controller	3	5	8
Other professions	2	0	2
**Total**	**17**	**24**	**41**
Function			
Head of clinic, CEO	2	4	6
Administrative staff	9	7	16
Working in different health professions	6	13	19
**Total**	**17**	**24**	**41**

The interviews were analysed using a thematic analysis.^36^ We adopted an abductive approach that allows for interaction with previous and newly discovered knowledge, thus allows for a combination of an inductive and deductive approach.^37^ Accordingly, the interview guide provided a helpful structure for beginning the analysis, but the themes were adapted when new aspects were discovered. The first step in the analysis was to identify what aspects of the new regulative framework healthcare providers experienced, how these aspects were perceived and whether they had any effect on their daily operations. Emerging themes were later sorted into the neo‐institutional framework by Scott,^28^ to connect the empirical findings to the conceptual framework based on institutional pillars. An iterative process followed, where identified themes were classified, grouped and regrouped. Only the quotes used in the article was translated from Swedish to English.

The originators of the quotes used in ‘Findings’ have been encrypted to ensure that individual respondents cannot be identified. The healthcare providers will be denoted A–D followed by a number indicating the respondent. Further, in the following text, post‐discharge care will be denoted as external care when provided by a provider other than the initial spine surgery clinic.

## 5 FINDINGS

The main themes correspond to the aspects of the STHLM‐VBRP that the healthcare providers experienced as most important: the bundled payment, the performance‐based payment, and the continuous contract. Each theme is followed by subthemes that were generated with the inductive approach.

### 5.1 The bundled payment

#### 5.1.1 A more holistic perspective

All healthcare providers were strongly affected financially by the increased cost responsibility. Hence, all healthcare providers experienced a strong incentive to take care of complications related to the surgery to avoid paying other healthcare providers. It further stimulated the providers to discuss how to decrease the amount of external care at other hospitals/providers.

What I think is the difference, when I look at other areas of patient choice, we have a more complete picture of the patient. Otherwise you only see the surgical procedure. So I think that is the major difference, that you have a lot more patient responsibility for much longer. It also creates other routines; it creates another way of working.C2

Yes, it actually drives private healthcare providers to take responsibility. Not just under the knife, but that you actually own the process for a while longer.B8

The invoices for external care created a new flow of information to the providers when their patients were treated elsewhere. Healthcare providers expressed that this procedure gave them a more comprehensive perspective of the care chain. They realised that not all patients contacted them if they experienced complications after the surgery.

That there is a slightly clearer follow‐up on how the patients turned out in the end. Because clearly patients may be a bit different, some may not always call you if they don't feel well. They think like ‘nah but I guess this is normal’, now we can see it in a different way – more clearly.D4

I think it's good that you have to take that responsibility because it also means that you are more active in taking care of your own complications. You have to because of financial reasons. If one of our patients ends up at a university hospital or something like that, then we get a huge invoice and its's not – it has happened – it's not funny. Like you see half a week's production just disappears. Yes, it's pretty tough but I still think it's good.C1

The financial responsibility for post‐discharge care affected healthcare providers. In particular, the cost of treating infections was extremely high and affected them greatly. Some respondents also said that the clinics in Stockholm all had very low infection rates. Thus, it was rather a matter of bad luck than something that was preventable and hence impossible to reduce further. Therefore, it seemed unfair to have them pay for necessary care for patients.

It is more that it feels unfair, when it comes down to a complication that you reasonably couldn't have avoided in any way, that you later receive a bill of half a million, it doesn't feel fair. It is more this sense of justice, you have done your absolute best and sometimes you get hit, this penalty approach is not good.D3

Thus, respondents expressed a level of discontent with the inclusion of infections in the responsibility of post‐discharge care. Infections requires intensive care, something the private spine surgery clinics cannot provide themselves.

#### 5.1.2 Post‐discharge care—An administrative nightmare

Another aspect of the increased financial responsibility was that invoices often seemed to include care that was not related to the spine surgery. This resulted in additional administrative work for administrators at healthcare providers, but also for physicians since it required medical knowledge to audit the health records of the patients. Respondents said that it would be impossible to keep auditing health records manually in the long term and emphasised the need of better IT‐facilitation for a sustainable system.

And should something happen after surgery that is related to the surgery – then of course, you have to take responsibility. The only thing that I find tiresome… is that they shift a lot of costs onto the healthcare provider that aren't related to the surgery. It's just that parenthesis, otherwise I definitely think you should be responsible for what you do, absolutely.D6

The unrelated care was experienced as an ‘unfair part of the contract’ (B7), a way for the purchaser to pass on costs to the provider. Another problem with unrelated care was that they had to spend time and resources on writing an appeal to argue as to why they should not pay the bill, thus increasing the administration even further.

#### 5.1.3 A more prominent role for physiotherapy

Due to the bundled payment the cost of physiotherapy became salient to the spine surgery clinics and acquired a far more central position as compared to before the introduction of the VBRP model. The close relationship between spine surgery and physiotherapy became more evident and it was discussed at a management level.

Now management can try to concentrate on how… before we've ignored physiotherapy. Now, we must include physiotherapy – what physiotherapy do we really need? And how should we work with it and to put those thoughts into practice.A1

Two of the healthcare providers perceived the more prominent role of physiotherapy as a natural step in improving spine surgery care. Healthcare professionals argued that it was good to assess patients from different perspectives, that physiotherapists can better assess the physiotherapy the patient has had previously.

We have a holistic perspective all the time because we have the reception, we have the surgery, we have the physiotherapists and in the city we provide – we are accredited within Patient Choice rehabilitation. So I think we have a holistic approach regarding the spine.A3

At the other two clinics it was experienced as a big transition from being a spine surgery clinic responsible for the patient from the surgery until discharge, to suddenly being responsible for post‐discharge care and rehabilitation. From 1 day to the next, physiotherapists suddenly had a great responsibility. One of the providers began to drastically change their patient flow by involving physiotherapists in the assessment but also by opening a centrally located outpatient clinic. Another provider had a more reserved attitude to the new responsibility making no major changes.

We have started up a completely new flow prior to surgery that we are implementing at the moment and slowly getting used to.B5

Yes, a little understanding would feel good because, as I said, referring patients to avoid things they actually need. […] I feel really sad about it and I think it's pretty difficult to work that way. It would be nice to have an understanding of why I say it … I do as I'm told but that doesn't feel that good all times.D8

Another problem was that it was not possible to assess what patients had been treated for by external physiotherapists after surgery. This caused frustration and the spine surgery clinics felt a lack of control. Respondents expressed the need for better registration of what kind of treatment patients received when consulting a physiotherapist.

We've experienced that patients who have had minor surgery, that may not really be in need of much physiotherapy, go to a physiotherapist in town and then loads of invoices drop into our mailbox and we can't figure out how that happened. We don't really have any control of the situation. But the patients do have the right to consult a physiotherapist, and then the physiotherapist can bill us.C1

The spine surgery clinic wants to be able to assess whether the treatment is related to the spine surgery or not, and the necessity of the treatment. Three out of the four clinics, offered the patients regular return visits to the clinic for physiotherapy, in an attempt to avoid being billed by external physiotherapists. This was also logistically problematic when patients lived far from the clinic and/or already had an established relationship with another physiotherapist. To increase the likelihood of the patient returning to the clinic for physiotherapy after surgery, one healthcare provider started with physiotherapy before the surgery to establish a relationship with an internal physiotherapist.

#### 5.1.4 Does increased accessibility and freedom of choice require a patient contract?

With an increased cost‐responsibility for post‐discharge care, respondents recognised that it was more important now to establish a good relationship with the patient. If the patient wanted to return to their practice, they would not have to pay other healthcare providers. To increase the chance of the patient returning for post‐surgery care, all healthcare providers had increased their accessibility by extending opening hours, two providers also opened up a more centrally located outpatient clinic.

We have better control and the patient has better access to us I think. So for the patient I think it's an advantage actually. And the most positive effect is probably that we feel that we must hold on to our patients, we lose out by not caring about them, I must say.A6

It was however logistically problematic for the providers to make patients come back in the case of complications, or to do physiotherapy when the easiest option for the patient was to turn to their primary care centre, emergency department or a physiotherapist with whom they already had an established relationship. This was described as unfair, to place this coordinating and integrating responsibility on them alone. They further expressed difficulties in scheduling staff; there is no point of having a physiotherapist at the clinic if the patients never use it.

The damned patients who don't come here.D7

As an effect, physiotherapy could be perceived as something that should not be recommended by healthcare professionals. They could not send patients in need of physiotherapy to other physiotherapists because of the financial responsibility.

But what we see is that the costs for physiotherapy are quite high because the patient still has a choice. We have no choice; we have signed this contract. The patient has a choice not to come here, where we already have the staff for that kind of activity.B8

But … we can't control patients, we can only ask them not to go, and if they should go, we want them to go to this physiotherapist with whom we have an agreement. But if they don't go there, we can't force them either. Because they have a free choice.D2

Many experienced it as frustrating not having any tools to handle the free will of the patient and wished for better support from Region Stockholm. Because of Patient Choice they could not limit the care the patient sought elsewhere. Some respondents suggested the possibility of a patient contract as a solution. A contract that guaranteed the patient a certain amount of physiotherapy related to the surgery, but the patient would be charged for any additional care that exceeds the agreed amount. However, that kind of action needs regulative support from Region Stockholm.

#### 5.1.5 Incentive for cooperation with other healthcare providers

Communication with external healthcare providers became important, but it was difficult to make them cooperate. Providers within STHLM‐VBRP acknowledged that there was no direct incentive for external healthcare providers to put time and resources into contacting them. However, from a wider perspective it would be more efficient since the spine surgery clinic has the clinical history of the patient. They meant it would be more efficient if they got the chance to take care of their own patients instead of using the resources of hospitals or other facilities dedicated to more serious or complex conditions. Respondents said that they had realised that they must work actively for more integrated care, to contact external healthcare providers to set up a dialogue, and together design optimal post‐surgery care.

In a way I think it's good. On the other hand, I think it's bad because you can't control if a patient chooses a provider other than yourself. Which makes it important to speak the same language with the external providers. You cannot force people to come back after discharge, they may think it's better to go to their own physiotherapist. And we can sometimes differ in the way we see things, how much care the patient needs.C3

The bundled payment made it important to build a network of external physiotherapists (and other healthcare providers), with whom the spine surgery clinics could cooperate and agree upon an adequate level of care.

Another problem respondents described was how to obtain information on whether their patient sought care elsewhere, and how to offer them the possibility to come back. Once they receive the invoice, it is too late to react, thus not giving them the chance to affect the healthcare provided. Respondents said that it felt like a punch in the stomach to receive an invoice from another hospital that had treated, a patient they could have treated themselves had they been offered the opportunity.

And it can be frustrating if it is abused out there, that someone gets loads of mediocre unnecessary care, I don't think that's okay. So we can try to have a good dialogue with them, to write referrals and write some guidelines and such.C3

Patients usually seek care at an emergency department and are hospitalised. And then all possible things can be done without anyone contacting us. They can perform surgery and they can do – we don't even know that the patient is there. And then the patient is hospitalised for two weeks or something, being treated, and different things are done, and then suddenly out of no‐where we receive a bill … it can cost a month's budget, for care we don't have any opportunity to influence.D1

Respondents said that some kind of automatic notification when their patient was registered elsewhere would be helpful, to give them a fair chance to offer their services. However, they also acknowledged the importance of respecting the choice of the patient and their option to say no and stay with the external healthcare provider.

### 5.2 The performance‐based payment

#### 5.2.1 The lack of financial impact

Healthcare providers experienced the performance‐based payment as something positive. The idea of being reimbursed based on results instead of activity, was encouraging. It was aligned with the professional values of ensuring the patient is free from pain 1 year after surgery. Respondents also expressed that they did not feel constrained by the reimbursement model or the performance measure used.

We have our own goals, but they coincide with the goal that we want to maximise for the patient – that they should be as well as possible. So I don't think we have different goals, rather that the financial goals are in line with the goal you have with the patient, so to speak.C2

Quality improvement was considered important and something they continuously worked with before the introduction of STHLM‐VBRP. All providers appreciated the idea of a performance‐based payment being used as a complement to the bundled payment. But it had no financial impact. The share of the performance‐based adjustment was too small in comparison to the prospective payment and the invoices for external care. Respondents meant that a larger proportion of the payment had to be tied to performance to generate an effect.

The performance‐based payment isn't anything we look at every month, in that way, unless it diverges a lot. It goes alright, and as long as our patients feel good we don't really follow‐up on it that intensely.D4

There is a certain idea behind it, so you have to make sure that the bonus, that the quality bonus works. Because otherwise, if you only take a part of it and don't care about the other part – then some can make as much money as they want while others can't. It's completely wrong! Why then would you do all this, without doing this part right? There must be an incentive there.A10

Some meant that the level or structure of the performance‐based payment had to be adjusted, otherwise the whole model would lose its purpose and might have negative effects on quality. Furthermore, the healthcare providers were not able to control the performance‐based payment they received from Region Stockholm.

#### 5.2.2 The lack of transparency in how the payment was calculated

Respondents were positive to the idea of the performance‐based payment but did not understand how they could make use of it. The lack of transparency for how the performance‐based payment was calculated made the model more difficult to understand. Respondents said that they had been promised a demo that showed how different variables affected the performance‐based payment. They had not yet received any demo but said that it would most probably be helpful to understand what aspects that affect the reimbursement level.

I agree with the idea – yes. But, then there's a lot that we don't really know so much about when it comes down to what matters when this performance‐based payment is calculated.A2

Because we want to understand, why did we get minus 15,000 and why did we get 10 for that? But then, it's so big that you cannot handle it. So you would want something just like this [snaps fingers], some kind of search engine.D2

The complication responsibility of post‐discharge care feels much more concrete, that there we can do something and we know what we're doing. Whereas this performance‐based payment, there we feel like we're just groping in the dark.A6

Compared to the bundled payment, respondents experienced the performance‐based payment as vaguer and more complex. Respondents said that the performance‐based payment was so low that it had no financial impact, it was not worth to monitor and assess outcomes measured with GA as a part of their daily operations.

#### 5.2.3 Concerns regarding potential shifts in case‐mix

Within elective spine surgery, it was experienced by the respondents that it can be difficult to tell if a patient will benefit from surgery or not. When relating the reimbursement to how much pain the patient experiences after a surgery, some respondents expressed a concern regarding potential shifts in case‐mix. The lack of effect of the performance‐based payment raised concerns regarding cherry picking. In the worst‐case scenario surgeons would only perform surgery on patients they knew for sure would benefit from it, thus not ‘taking a chance’ with patients suffering from comorbidities. At the same time, they argued that they should not perform surgery on patients who would not benefit from it, thus recognising the complexity of the problem.

Obviously, when you do more risky things, you know that you take a greater medical and financial risk. Because if the surgery fails we must face the consequences. And of course, in the worst case scenario Patient Choice could lead to some patients being excluded. Then personally, I don't think I actually do that, but theoretically it could absolutely be possible. Especially if you have a lot of patients, but if you only have a few patients obviously you can't turn them away. If you have a lot of patients, then cherry‐picking may be a problem.C1

However, when the fourth provider entered the market the competition increased, and no provider could afford to say no to patients. Providers focused on how to build processes that decreased the need of post‐discharge care and physiotherapy. Respondents said that due to the extended cost‐responsibility, the reimbursement level was relatively lower compared to before and this gave incentive to increase production. Thus, the decreased reimbursement level in combination with an increased competition, prevented cherry picking according to respondents.

### 5.3 The continuous contract

#### 5.3.1 A decreased level of uncertainty allows for long‐term planning but with a diminishing reimbursement level

The continuous commissioning contract allowed accredited healthcare providers to make long‐term plans because they did not have to fear losing the contract during a competitive procurement process.

Because of Patient Choice, we got more freedom to be able to decide ourselves what we think will be the best for the patient. That made us realise immediately that we must do something preoperatively and postoperatively.A7

Providers expressed frustration with the inability of the purchaser to monitor and assess the quality of the healthcare provided. Respondents meant that this inability caused a competition based on price, regardless of quality under procurement.

I've been involved in quite a few procurements and in the end it's only the price that matters. And there are many actors who are not serious, who don't take on the most difficult or weighty procedures, more difficult patients and put in really low bids during the procurement process. A serious clinic can't practice under those circumstances.A1

All providers had a positive attitude to competition based on quality instead of price. However, they raised concerns regarding the lack of adjustment to the price level in line with inflation. Region Stockholm did not adjust the reimbursement level during the first 4 years and respondents doubted that this was going to happen at any time in the near future.

It's the price erosion that I'm worried about. When there is no adjustment to inflation, and the margins get to the level that we must begin to reduce the quality. So that is the most important question right now.A1

Even though all healthcare providers agreed that the reimbursement level was at reasonable level for spine surgery procedures, it was relatively low in relation to the cost for post‐discharge care.

We might be more effective because we have had to make some cuts. We have had to assess our working routines, and that's not only negative. The coin always has two sides, so it can actually be positive as well. But it has been difficult.B9

One healthcare provider expressed concern regarding the new level, fearing that it would eventually affect patients negatively. Although they admitted that this new level had forced them to make changes that were for the better, but they feared that it would not be enough. Especially without future adjustment of the reimbursement to inflation.

## 6 THE ALIGNMENT AND MISALIGNMENT BETWEEN REGULATIVE, NORMATIVE, AND CULTURAL‐COGNITIVE ELEMENTS

In Table 2, we summarise the alignment and misalignment between the regulative, normative, and cultural‐cognitive elements among healthcare providers.

**TABLE 2:** The findings in relation to the neo‐institutional pillars, (+) indicates that the aspect aligns with the STHM‐VBRP, whereas (−) indicates resistance to the STHLM‐VBRP

Regulative	Normative	Cultural‐cognitive
The bundled payment		
Increased financial responsibility for post discharge care.	**(+)** New identity, not only a spine surgery clinic. Must handle care‐flows outside their clinic.	**(+)** A new way of thinking, requires creative solutions. No shared logic of action.
Invoices for external care.	**(+)** High financial impact, incentive to coordinate post‐discharge care and discuss physiotherapy at a managerial level.	**(−)** Difficulties cooperating with other providers. It is beyond their conceptual world; do not have the right tools.
	**(−)** Reviewing and disputing invoices is time consuming for both administrative and clinical staff.	
Information about post‐discharge care.	**(+)** Increased focus on the relation between provider and patient resulted in better accessibility.	**(−)** The logic ‘the customer is always right’ is not aligned with the professional logic and agency.
No authority to impose sanctions on other healthcare providers.	**(−)** Spine surgery clinics have no authority to sanction undesired behaviour.	**(−)** No constitutive schema.
Vague definition of related care in the responsibility for post‐discharge care.	**(−)** Allows for different interpretation of related care. Unfair strategy by Region Stockholm, decreases trust between provider and purchaser.	**(−)** Confusion. Long lead times on disputed invoices increase uncertainty and obstruct providers when developing new processes to handle post‐surgery care.
	**(+)** Something Region Stockholm cannot sort out. As a provider of care, they are one of the best suited to solve the problem.	**(−)** Confusion because of vague definition of responsibility.
		**(−)** No constitutive schema or mental scripts.
The performance‐based payment		
Reimbursement based on patient reported outcome measure 1 year after surgery.	**(+)** Aligned with professional values, no constraining effect on perceived autonomy.	**(+)** Perceived as an innovative step by the purchaser, proof that quality matters.
Healthcare providers only perceived the performance‐based adjustment, not the full payment.	**(−)** The adjustment had no financial impact. Instead, providers focussed on how to decrease post‐discharge care that had a more direct impact.	**(−)** No difference with or without the performance‐based payment, they were already working towards good outcomes.
Healthcare providers did not receive information of how the payment was calculated for each patient.	**(−)** The performance‐based payment was perceived as too complex. It was not worth the effort to understand it better.	**(−)** Confusion caused by the lack of transparency in how the payment was calculated.
The continuous contract		
A continuous contract between provider and purchaser.	**(+)** The decreased uncertainty allows healthcare providers to make plans for the long term.	**(+)** Increased autonomy. Providers can design their own processes.
A set price without adjusting the price level in line with inflation.	**(+ −)** Quality enhancing but the lack of adjustment makes providers uncertain about the purchaser's intentions.	**(−)** Healthcare providers still perceive Region Stockholm to put price before quality.
No restrictions on volume.	**(+)** The providers can assess patients without any restrictions, everyone in need should be treated.	**(+)** More information on which patients benefit from surgery is needed.
The importance of support and communication with the purchaser.	**(+)** More information, give control to providers to be able to follow‐up on care.	**(−)** No constitutive schema to handle information.

The bundled payment implied a new way of thinking about elective spine surgery. The idea of taking a greater responsibility for the care chain and increase cooperation among providers was supported by all three pillars. However, how to act on these ideas was not obvious to healthcare providers. As mentioned in section two, confusion usually indicates a lack of support from the cultural‐cognitive pillar. Especially how to better integrate physiotherapy and increase cooperation with external healthcare providers. To know how to act they ‘must learn about the new contract and gain an understanding of it’ (B7). Without a shared understanding they cannot find appropriate means of how to adapt their practice. Despite efforts to cooperate with external healthcare providers, it was impossible to reach all of them and establish consent regarding optimal medical practice. Respondents experienced the situation as rather hopeless without any authority to impose sanctions if care, in their opinion, deviated from optimal medical practice. Since respondents experienced cooperation with other healthcare providers as difficult, they discussed a patient contract as a potential solution by holding patients accountable for excessive rehabilitation. Hence, this hopelessness resulted in new normative and cultural‐cognitive values that lacked regulative support. New normative and cultural‐cognitive values should be taken into consideration by the purchaser when updating the reimbursement programme.

The definition of related care is important since it affects the range of healthcare providers' responsibility. Because of the vague definition, the responsibility could be perceived as both narrow and wide. Healthcare providers that adopted a narrower definition, experienced invoices pertaining to unrelated care as something unfair. It affected their relations with the purchaser because they felt used and experienced it as an unfair strategy. On the other hand, healthcare providers that adopted the wider definition of related care had a more neutral perspective on the invoices from Region Stockholm. However, the experienced ambiguity resulted in healthcare providers not knowing how to design their processes, since they did not know to what extent they were responsible. The vague definition in the contract weakened the regulative pillar. Due to the fact that providers did not know how to assess related care nor how to act on it, there was no support from the cultural‐cognitive nor the normative pillar to strengthen the regulative pillar. Hence, the vague definition weakened the institution by motivating different behaviours.

Providers appreciated the performance‐based payment and perceived it as a quality statement by the purchaser. To reduce the pain further than expected, it is crucial for healthcare providers to come to an understanding of what else that can be done in addition to surgery. For a spine surgery clinic, this can be challenging since their specialisation in fact is spine surgery. Hence, the payment incentivise interprofessional collaboration and holistic healthcare since surgery alone may not be enough to reduce the pain any further. However, healthcare providers received no information about the expected performance‐based payment nor the patient's expected pain reduction. The lack of transparency in how the performance‐based payment was calculated made the payment too complex to understand, and because of the lack of financial impact, it was not worth trying to understand. The intended incentives to improve quality failed to have impact on behaviour since it lacked support from the cultural‐cognitive pillar (they did not understand it) and the normative pillar (it was not worth understanding). Because of the strong financial impact of the bundled payment, healthcare providers focussed on how to minimise costs rather than maximising their outcome regarding the performance measure.

The continuous contract with no restriction regarding volume was supported by all three pillars; it decreased uncertainty to healthcare providers and increased their autonomy. Respondents did however express the need of more research to better distinguish the patients that ultimately benefit from surgery and what kind of physiotherapy that is motivated. The idea of a set price to promote competition based on quality was aligned with the normative and cultural‐cognitive pillars of healthcare providers. However, respondents experienced that the lack of adjustment of the price level in line with inflation was undercutting quality and the existence of their practice. As one respondent (B7) put it ‘they squeeze every penny out of us making it really hard for us to survive as a business’. This caused normative resistance because respondents experienced it as unfair, and it seemed like Region Stockholm still put price before quality.

Our analysis showed that the value‐based reimbursement programme caused misalignment between the institutional pillars among healthcare providers. Even though providers supported the general idea of the value‐based reimbursement programme, how to act on these ideas and adapt their practice to it required comprehensive understanding of the programme.

## 7 DISCUSSION

The bundled payment imposed strong financial incentives thus making it crucial for all healthcare providers to assess the whole care chain of their patients. Due to the responsibility, healthcare providers became more prone to strive for optimal healthcare consumption. Similar findings have been reported in other studies, where bundled payment reduced healthcare use and costs.^21^, ^38^ The different perceptions, of the legitimacy of increasing healthcare providers responsibility for post‐discharge, is in line with studies showing that spine surgeons allocate major responsibility to healthcare systems to manage the cost of healthcare.^39^ With STHLM‐VBRP, Region Stockholm moves towards integrated care with patient‐centeredness and a greater responsibility to healthcare providers, thus in line with VBHC.^40^

Participants in our study experienced a need for better cooperation between healthcare providers. Similar findings were reported in a study that investigated the effects of introducing VBHC in another Swedish region.^41^ They found that the introduction of VBHC raised awareness about cooperation being necessary to create value for patients. The introduction of VBHC increased cooperation within the hospital but it was difficult to establish cooperation with external healthcare providers.^41^ In the case of STHLM‐VBRP, the private healthcare providers experienced difficulties in making other providers cooperate when there was no corresponding incentive for external providers. Thus, insufficient organisational structure of healthcare hinders structural changes.^42^ The difficulty in establishing cooperation reflected that health care providers did not have cognitive schemas of how to coordinate post‐discharge care nor how to act on the information they received from the invoices of external care and rehabilitation. Insufficient support from purchaser bodies (governmental or private) have been identified as a barrier for institutionalisation of elements of VBHC (such as VBRP) across different healthcare systems.^23^ Thus, better dialogue between purchaser and providers in combination with compatible and agile IT‐systems may enhance cooperation. The purchaser has to facilitate cooperation between healthcare providers to make the cost of cooperation as low as possible. The struggle of healthcare providers does on the other hand show that patients have a free choice to choose whomever they prefer.

The performance‐based payment has been criticised to have a negative impact on some aspects of medical professionalism.^43^ Participants were positive to the performance‐based payment because it did not ‘force’ them to focus on irrelevant outcome measures. However, the lack of transparency and financial impact made the performance‐based payment too complex to understand and follow‐up. Quality improvement requires ongoing feedback at all stages, and that everyone involved is aware of the complexity of changing the culture of the organisation.^42^, ^44^ The slow feedback from Region Stockholm caused frustration among healthcare providers. It made it more difficult for them to adjust to the new structure.

Participants in our study found that their focus on financial aspects had increased with the STHLM‐VBRP, compared to before. A potential explanation can be the experienced unbalanced incentive structure between the bundled payment and the performance‐based payment. Thus, healthcare providers focussed on minimising post‐discharge care instead of quality improvement, contrary to other studies where VBHC decreased the focus on financial aspects.^41^ It has been argued that financial incentives are not sufficient to affect daily operations in a setting where governance and management philosophies are firmly grounded within the New Public Management paradigm.^16^ We argue that financial incentives are, if introduced in the right institutional context, an effective tool to manage care. This highlights the importance of acknowledging the institutional context when designing, implementing, and evaluating reimbursement programmes.

Another important aspect was the transition from periodic re‐contracting to a continuous commissioning contract, which according to respondents decreased uncertainty of the future and allowed them to make long‐term plans. However, it was also clear that a continuous contract requires relevant feedback from the purchaser at the right time. Once again, this highlights the importance of good communication and supporting IT‐systems. Especially since the text in the commissioning contract can be interpreted in different ways (as the vague definition of related care proofed), therefore the relation to the purchaser organisation is more than just a commissioning contract.

Even though this study was performed in a context of Swedish healthcare, it investigates management practices that are globally diffused and therefore should be of relevance for other healthcare contexts as well. Despite differences in healthcare organisation and funding between healthcare systems across the world there seem to be universal enablers and barriers for implementation of VBRP. Based on a comparison of four different healthcare systems, Mjåset et al.^23^ found that three aspects were universal for a successful implementation: (1) strong support from governmental/purchaser bodies, (2) IT‐systems that allow seamless system integration and up‐to‐date outcome measurement across the full cycle of care, and (3) involvement of the medical community to make sure that the intrinsic values of working in healthcare are aligned with management strategies. These aspects are further manifested by our findings from studying the introduction of the STHLM‐VBRP.

Because of contextual differences between and within healthcare systems, it is important to use a theoretical framework to structure findings and enable comparison.^27^ We included all four healthcare providers that were reimbursed based on the STHLM‐VBRP. Despite the limited number of providers, they performed a majority of the elective spine surgeries in Sweden. However, by that limited number, we cannot claim to cover all experiences. To cover other perspectives from the same reimbursement programme, future studies could focus on how the purchaser organisation act and adapt their practice to a value‐based reimbursement programme. It would also be of great interest to study the reimbursement programme in a context that involves more providers.

The introduction of STHLM‐VBRP had three defining features: the bundled payment, the performance‐based payment and the continuous contract between provider and purchaser. The general perception among providers about these features was positive. However, our analysis showed that the resistance to STHLM‐VBRP was mainly caused by confusion in how to interpret and act on the information they received, that is, misalignment with cultural‐cognitive pillar. The misalignment between the institutional pillars in healthcare providing organisations can be seen as a catalyst for change because of instable institutions. Whether this change will lead to more robust institutions depends on whether healthcare providers can come to an understanding of how to coordinate post‐discharge care, have access to sufficient IT‐systems, and whether the purchaser is able to support the healthcare providers when taking these steps towards integrated healthcare.

## CONFLICT OF INTEREST

The authors declare that they have no conflict of interest.

## ETHICS STATEMENT

Before commencing the fieldwork, the study obtained ethical approval (2015/94‐31) from the regional board of ethics in Linköping, as well as a signed consent to participate from each respondent.

## Data Availability

The data generated and analysed during the current study are not publicly available due to the inclusion of potentially sensitive individual data. The ethical approval includes a statement that the data will be kept in a private repository but are available from the corresponding author on reasonable request.
